# Effect of Diabetes Mellitus on Tuberculosis Treatment Outcome and Adverse Reactions in Patients Receiving Directly Observed Treatment Strategy in India: A Prospective Study

**DOI:** 10.1155/2016/7273935

**Published:** 2016-08-24

**Authors:** Ali Nasir Siddiqui, Khalid Umer Khayyam, Manju Sharma

**Affiliations:** ^1^Department of Pharmaceutical Medicine, Faculty of Pharmacy, Jamia Hamdard, New Delhi 110062, India; ^2^Department of Epidemiology & Public Health, National Institute of TB and Respiratory Diseases, New Delhi 110030, India; ^3^Department of Pharmacology, Faculty of Pharmacy, Jamia Hamdard, New Delhi 110062, India

## Abstract

Despite successful implementation of directly observed treatment, short course (DOTS) in India, the growing number of diabetes mellitus (DM) patients appears to be a cause in the increasing tuberculosis (TB) incidence, affecting their management. In this regard, a prospective study was conducted on DOTS patients in three primary health care centers in urban slum region of South Delhi, India, to evaluate the effect of DM on sputum conversion, treatment outcome, and adverse drug reactions (ADR) due to anti-TB treatment. Eligible TB patients underwent blood glucose screening at treatment initiation. Disease presentation, clinical outcome, and ADRs were compared between patients of TB with and without DM. Out of 316 patients, the prevalence of DM was found to be 15.8%, in which 19.4% and 9.6% were PTB and EPTB patients, respectively. DM patients have observed higher sputum positivity (OR 1.247 95% CI; 0.539–2.886) at the end of 2-month treatment and poor outcome (OR 1.176 95% CI; 0.310–4.457) at the completion of treatment compared with non DM patients. Presence of DM was significantly associated (OR 3.578 95% CI; 1.114–11.494, *p* = 0.032) with the development of ADRs. DM influences the treatment outcome of PTB patients in our setting and also on the ADR incidence.

## 1. Introduction

The bidirectional association between tuberculosis (TB) and diabetes mellitus (DM) is currently one of the major concerns for clinicians, as DM affects the disease presentation and clinical outcome of TB and vice versa [1]. This comorbidity is known since the beginning of the 20th century. However, recent increase in the number of DM patients, attributed mainly to the modern lifestyle changes, created interest to further assess the association between both diseases [2]. The coepidemic is emerging predominantly in resource poor countries where the burden of DM is increasing and also TB is highly endemic [3].

The prevalence of DM in India is rising and estimated to reach 123.5 million by 2040 [4]. India ranks the highest in TB burden with 23% of the global incidence cases in 2015 [5]. Active TB and reactivation of latent infection have long been known to be a risk of DM. A recent systematic review demonstrated approximately 3 times higher risk of developing TB in DM patients than no-DM patients [6]. TB infection also deteriorates the glycemic control and reduces the effectiveness of DM management [7]. Multiple studies from different countries reported 12%–44% of TB cases linked with DM at the time of TB diagnosis [8–12]. The patients of pulmonary TB with DM experienced poor rate of sputum conversion at the end of 2-month regimen along with higher rates of treatment failure and deaths at the end of treatment as compared to no-DM patients [13–15]. Fewer South Indian studies have reported higher DM prevalence but scarce data is available from Northern India [8, 9, 11].

The anti-TB therapy includes a long-time, wide spectrum of drugs, which can predispose patients to develop adverse drug reactions. The emergence of adverse reaction depends on the patients' characteristics and also on concomitant medication during therapy [16]. The use of anti-DM medication may lead to interactions with antitubercular drugs. A subjective assessment is therefore essential to elucidate the factors associated with anti-TB medication adverse reaction, which may determine adherence and, therefore, therapy success.

In this underlying work, we report some of the information gaps that have been recognized on the association between TB and DM, particularly from North India [17]. A systematic assessment is needed as the merging epidemics, especially in low- to middle-income countries, are experiencing the fastest increase in DM prevalence with highest TB burden [18]. Paucity of literatures and lack of awareness increase in the challenge of the management of such patients as the burden of DM is uninterruptedly rising. The objective of present study was to describe the disease presentation, sputum conversion, treatment outcomes, and adverse drug reaction (ADR) incidences in patients of TB with and without DM.

## 2. Methods

### 2.1. Study Population

Three outpatient primary healthcare centers (PHC), namely, Mehrauli, Khanpur, and Tigri, were selected from South Delhi, India, for patient enrollment from January 2014 to September 2014. These PHCs are affiliated to a tertiary institute, that is, National Institute of TB and Respiratory Diseases (NITRD). All PHCs were located in the urban slum part of South Delhi. TB was diagnosed on the basis of clinical presentation and was confirmed by microscopic detection of acid-fast bacilli (AFB).

### 2.2. Ethical Approval

This study was approved by the ethics committees of Hamdard University and National Institute of TB and Respiratory Diseases (NITRD/EC/2014/10293), New Delhi, India. Written informed consent was obtained from all subjects before the patient enrollment.

### 2.3. Study Design and Sample Selection

The new and retreatment TB patients above 15 years of age and attending directly observed treatment, short course (DOTS) clinics at selected PHCs were undertaken in this prospective study. Eligible patients included those of category I (new cases of sputum smear positive, sputum smear negative, extra pulmonary tuberculosis, and other cases) or category II (retreatment cases of recurrent TB, treatment after failure, treatment after loss to follow-up, and other previously treated patients) were considered for this study. Patients below 15 years of age, suspected or known multidrug resistance (MDR) TB patients, and those who were not willing to participate were excluded from the study. In addition, patients diagnosed with any disease other than TB and DM were also excluded to avoid the confounding effect on treatment outcome. In this study, the proportion of TB patient was estimated to be 25%. We calculated sample size at 95% confidence interval for proportion *p* with margin of error *d* according to the formula: *n* = (1.96)^2^ *pq*/*d* ^2^, where *q* = 1 − *p*. (1.96)^2^ × 0.25 × 0.75/(0.05)^2^ = 288. With an estimated 10% loss to follow-up, a total of 316 patients were enrolled in this study from all three centers.

### 2.4. Measurement of Glucose Concentration

All enrolled patients were screened for fasting blood glucose (FBG) at TB treatment initiation. Those whose FBG was found beyond 110 mg/dL were repeated for 2-hour plasma glucose (2 h PG) after 75 g oral glucose tolerance test (OGTT). DM was diagnosed if the 2 h PG was found ≥200 mg/dL in accordance with international criteria [19, 20]. Prediabetes patients were not included in DM category. Finally, TB patients were classified into two groups, one with DM and another with no DM, based on their DM status. Newly diagnosed patients were referred to local PHC physician for DM management, and details were recorded in predefined record sheets. The prescription details and ongoing medication were also recorded for known DM patients.

### 2.5. Collection of Covariates and Other Symptoms

A pretested, semistructured questionnaire was designed to collect information on sociodemographic profiles, clinical presentation, and signs and symptoms at treatment initiation. Additionally, status of DM with their management, previous TB treatment history, treatment results, medications, duration of DM, and outcome of therapy were recorded in standardized data collection sheet. Sign and symptoms were calculated in a score of 1 to 3, with lower numbers reflecting the higher severity of symptom. Presence of cough, weight loss, evening fever, anorexia, dyspnea, chest pain, and hemoptysis was recorded by face-to-face interviews. Patients with a composite score of 07, one for each symptom, were classified as being highly symptomatic. Disease severity was evaluated by sputum mycobacterial load. Sputum sample of PTB patients was subjected to microscopic examination of Ziehl-Neelsen staining and was performed for acid-fast bacilli [21]. Mycobacterial load in the sputum was graded as +, ++, or +++. Patients were followed up for repeat sputum examination at the end of intensive phase (IP) at 2 months and at the completion of treatment.

Following the World Health Organization (WHO) standard regimen guidelines under Revised National TB Control Program (RNTCP), treatment initiation of newly diagnosed cases was started with four drugs in IP for two months followed by two drugs in continuation phase (CP) for four months (2HRZE/4HR). Retreatment cases were initiated with five drugs in IP (2 months) followed by three drugs in CP for five months (2HRZES/1HRZE/5HRE). Treatment outcomes were defined as per the operational definitions of the program as per WHO guidelines (Table 1) [22].

### 2.6. Adverse Drug Reaction (ADR) Monitoring

The ADRs were recorded in the suspected adverse drug reporting form, that is, “voluntary reporting of adverse drug reactions by healthcare professionals.” Researcher has immediately recorded when any adverse reaction emerges, and routinely patients were closely supervised until completion of anti-TB medication.

ADR was defined as a response which is noxious, unintended, and occurs at doses normally used in humans [23]. Serious adverse events (SAE) are any untoward medical occurrence that, at any dose, is life-threatening or results in hospitalization or prolongation of existing hospital stay, persistent or significant disability, or death [23]. We considered liver dysfunction after an increase in serum alanine aminotransferase (ALT), or total bilirubin greater than two times the upper limit of normal (ULN), irrespective of the symptoms, in our study [24]. According to American Thoracic Society (ATS) guideline, ALT elevation more than three times the ULN in the presence of hepatitis symptoms and/or jaundice or five times the ULN in the absence of symptoms needs interruption and, generally, a modified regimen is used [25]. Except liver dysfunction, other ADRs including rashes, peripheral neuropathy, joint pain, gastrointestinal disorder, and others were determined based on symptoms/clinical examination as well as medical records.

In case of identification of suspected ADRs, the patients were followed up until resolution or end of TB therapy. Severity of ADRs was symptoms-based, as mild reaction shows no immediate modification of the standard regimen, and moderate reaction may require preventive measures, interruption, dose reduction, drug replacement, and discontinuation of anti-TB drugs [26]. Add-on medication includes antiemetic for relieving minor gastrointestinal reactions (nausea or vomiting) or an antihistaminic agent for reducing minor allergic reactions being manifested as itching. However, generalized erythematous rash, associated with fever and/or mucous membrane, lead to discontinuation of all drugs immediately [27]. Philadelphia tuberculosis control program suggested discontinuation of the drugs if skin reaction appeared till it gets resolved. Further, identification of the causative agents is recommended by rechallenging each drug [28]. Dosage of pyrazinamide and/or ofloxacin should be reduced or the drug withheld temporarily, if arthralgia is not responding to NSAIDs. In renal impairment, the dose of aminoglycosides may be reduced or replaced with other potent nonnephrotoxic antituberculosis drugs. Further, dose adjustment is required with ethambutol, quinolones, cycloserine and PAS in presence of mild to moderate renal impairment [29].

### 2.7. Statistical Analysis

Data collected were analyzed using Statistical Package for Social Science (SPSS)* version* 21.0. The finding of patients having TB with DM was compared with those of TB without DM. Categorical variables were compared by chi square test and continuous variables by Student's *t*-test. PTB patients were classified further as sputum positive or negative patients. Odds ratio (OR) was determined for sputum conversion and treatment outcome in DM and non-DM TB patients using logistic regression analysis. Risk factors associated with sputum conversion and treatment outcome were assessed by multiple logistic regression. Patient age, sex, body mass index, TB history, habitual risk, and clinical presentation were included as independent variables in logistic regression models. Further, factors influencing the anti-TB ADRs were analyzed by logistic regression. A two-tailed *p* value of <0.05 was considered significant.

## 3. Results

### 3.1. Patient Characteristics

**Figure 1:**
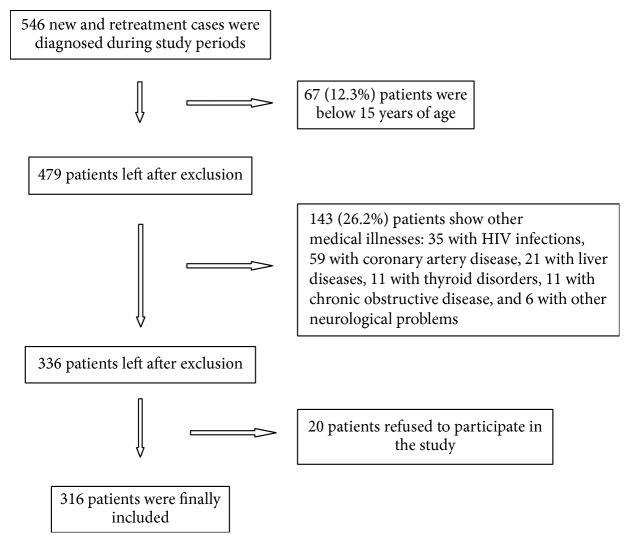
Flow diagram of study participants.

A total of 546 new and retreatment cases were diagnosed and subsequently managed at all three DOTS centers between the study periods. The patients who fulfill the inclusion criteria were recruited for this study. The flow diagram of subject inclusion is depicted in Figure 1. We recruited 316 patients with PTB and EPTB of both categories I and II during the study period from respective DOTS centers. Among all patients, 191 (60.4%) were diagnosed with PTB and 125 (39.6) with EPTB. Out of these, 15.8% (50/316) were diagnosed with DM, of which 9.49% (30/316) were diagnosed prior to TB diagnosis and the remaining 6.33% (20/316) at the time of DM screening at treatment initiation. The DM was more in PTB patients (19.4%) as compared to EPTB patients (9.6%). A comparison of patients with and without DM is depicted in Table 2. Our result shows that DM patients were more likely to be male, be of older age, and have higher mean BMI. The significant proportion of TB appeared to increase with age in DM patients compared to non-DM patients. However, both groups showed similarities in terms of sex, religion, family history of TB, and type of TB cases (new or retreatment).

Majority of patients in both groups have shown common TB symptoms. Patients with DM presented with more symptoms of dyspnea, chest pain, and hemoptysis while the remaining other symptoms including cough, weight losses, anorexia, and evening fever were predominant in non-DM patients as presented in Table 2. Newly diagnosed DM patients were confirmed to be type 2 DM, while among known DM patients, except one all belong to type 2 DM category. Regarding the management of DM with hypoglycemic agents, we found that common medication includes biguanides (40%), sulphonylureas (24%), insulin (08%), thiazolidinedione (02%), and gliptins (02%). However few patients (06%) were receiving herbal medicine for DM management. Four patients (08%) underwent only dietary management and 18 (36%) were advised for exercise also.

### 3.2. Treatment Outcomes

Of the total 191 PTB (31 and 6 were smear positive and smear negative in DM group, resp., and 109 and 45 were smear positive and smear negative in non-DM group, resp.) patients, 177 (92.7%) had completed treatment while 18 (4, loss to follow-up; 10, died; and 4, treatment regimen changed) did not complete it. However all EPTB patients have successfully completed the treatment. 232 (73.4%) patients initiated treatment within 07 days of their diagnosis and all received DOTS regimen. The sputum conversion and treatment outcomes of PTB patients during and at the end of treatment were shown in Table 3. Higher proportions (5.6%) of DM patients were lost to follow-up during the course of treatment but none experienced treatment regimen change or multidrug resistant TB (MDR-TB) compared to non-DM patients (2.7%) (Table 3). As previously reported, 10 patients died during the treatment of which one (2.8%) belongs to DM and the remaining 9 (6.0%) belong to no-DM group. Before completion of IP, one patient died in DM group, while 3 died and 1 was diagnosed with MDR in no-DM group. Microscopic examination of sputum samples at 2 months reveals higher sputum positivity in DM (27.8%) as compared to no-DM (24.7%) patients. Logistic regression analysis showed that DM with TB patients had a higher probability of delayed sputum conversion (OR: 1.247, 95% CI: 0.539–2.886) and poor treatment outcomes (OR: 1.176, 95% CI: 0.310–4.457) as compared to no-DM patients (Tables 3).

The association of different variables with the treatment outcome has been presented in Table 4. A statistically significant difference was obtained in the TB category, types of TB, and smoking history in the outcome analysis. Baseline clinical characteristic such as fever, dyspnea, and chest pain were also significantly associated with the treatment outcome. In Table 5, all independent variables were examined in multiple logistic regression analysis to find the association of these variables and sputum conversion >60 days and poor treatment outcome at the end of treatment.

### 3.3. ADR Incidence

A total of 224 patients presented with at least one ADR (224/316, i.e., 70.9%), of which 178 (178/266, i.e., 66.9%) had no DM and 46 (46/50, i.e., 92.0%) had DM. The median duration (±SD) between onset of anti-TB treatment and first-time adverse reaction occurrence was 14 (±14.63) and 14 (±14.06) days in DM and no-DM group, respectively. DM patients were commonly encountered with restlessness (42.0%), peripheral neuropathy (36.0%), liver disorder (22.0%), rashes (18.0%), and other nervous system disorders (40.0%). Other frequent ADRs that have been experienced were nausea, vomiting, arthralgia, drowsiness, and pain in back and limbs (Table 6). All ADRs were mild to moderate. Rechallenge of suspected drug was not performed in most cases due to safety and practical necessity. Among the collected ADRs, restlessness, hypoglycemia, back pain, and feet pain were significantly associated with TB with DM patients. The occurrence of other ADRs was not significantly different between the two groups as presented in Table 6. Upon subanalysis of ADRs according to the TB category, we found similar ADRs (restlessness, hypoglycemia, back pain, and feet pain) to be significantly more associated with DM patients in both categories I and II. However, no ADRs were significantly different while comparing DM patients of category I and category II as described in Table 7. Further, this study revealed that 53 patients (16.8%) appeared with one, 63 (19.9%) with two, 53 (16.8%) with three, 33 (10.4%) with four, 11 (3.5%) with five, and 10 (3.2%) with more than five ADRs, among 224 cases. The number of ADRs among DM and no-DM patients is given in Table 8. Our analysis also demonstrated that DM patients were encountered with significantly higher number of ADRs as compared to non-DM patients. The frequency of 5 or more ADR incidences was also significantly higher in DM patients (*p* ≤ 0.001).

We have further found that 4.43% (14/316) of total patients required modification in their anti-TB treatment due to ADRs. Most of these modifications were in the form of add-on therapy, where antihistamines and antiemetic were prescribed by the clinician. Few were also prescribed with pyridoxine to avoid the peripheral neuropathy. Occurrence of side effect was associated with being male (OR, 2.013 95% CI: 0.906–4.473), being in category I (OR, 2.165 95% CI: 1.004–4.670), having PTB (OR, 1.071 95% CI: 0.555–2.065), being married (OR 1.618 95%: 0.804–3.258), being Hindu (OR 1.131 95% CI: 0.498–2.567), and having DM (OR 3.578 95% CI: 1.114–11.494) in multivariate analysis. Except category I (*p* = 0.049) and DM (*p* = 0.032), no other variables were significantly associated with ADR incidence as presented in Table 9. The odds of developing ADRs were 3.5 (OR; 3.574; 95% C.I; (1.114–11.494)) times higher in DM than no-DM patients.

## 4. Discussion

Despite the evidences of concurrent increase in the incidence of TB and DM cases, there is very limited data available from north Indian population presenting the association of this comorbidity. The Union/World diabetes foundation has acknowledged the need of more epidemiological research to determine the TB burden attributed to DM, particularly in developing countries. The present work highlights the consequences of DM on the disease presentation, treatment outcome, and ADRs of anti-TB medication.

The overall prevalence of DM in our study was found to be 15.8% which is well above the global DM prevalence (9.0%) among general population [30]. Similar DM occurrence among TB patients has also been demonstrated in other tropical countries [31, 32]. In previous literatures, a wide range of DM prevalence from 1.9% to 35% has been reported among TB patients [1]. Further, we have recorded 40% newly diagnosed DM patients; probably they remain unrecognized due to delay in DM screening [33]. Few studies from Tanzania and Indonesia have reported 73% and 61% of newly diabetics diagnosed concurrent with active TB, respectively [10, 34]. This again confers the need of expanded medical attention in relation to DM screening and its management for improvement of TB treatment outcome.

The data in this study showed that PTB patients with DM have reduced rate of sputum conversion with higher probability of poor treatment outcome, namely, default, death, failure, and shifting to MDR category, than patients without DM. Consistent with the previous studies, we have also found more severe clinical manifestation among patients with TB and DM [15, 35]. The finding regarding sputum conversion at the end of 60 days also agrees with other studies [17]. The independent risk of poor outcome among PTB patients associated with DM in our study was 1.176 (95% CI: 0.310–4.457), which is little lower than the previously reported pooled risk of 1.69 (95% CI: 1.36–2.12) associated with the TB treatment failure and death [17]. This difference may be attributed to relatively smaller sample size in our study.

Analyzing symptoms associated with TB, compared to non-DM patients, weight loss was more frequent in TB patients with DM. Though weight loss is associated with both TB and DM, we have found relatively lesser weight loss in DM patients in our study. Contrary to this, Alisjahbana et al. (2010) have reported more weight loss in DM patients [10]. Few authors have revealed that clinical characteristics of TB do not differ among DM and non-DM patients [33, 35]. In agreement with others, we have also found less extrapulmonary involvement among DM than in no DM patients [33]. Contradictory reports are found with reference to the rate of positive smears at the time of diagnosis to different populations. We have observed higher positive smears among DM patients (22.6%) as compared to negative smears. Alisjahbana et al. (2007) reported a higher frequency of negative sputum smears among DM patients [10], while few showed no association between DM and sputum status of patients [36].

Current literatures on the effect of DM on sputum conversion are also conflicting. The independent risk of sputum positivity, associated with DM, is 1.176 (95% CI: 0.310–4.457) at the end of 60 days in our study. Few studies did not reveal any relation between DM and sputum conversion rate at the end of 60 days [1, 14]. Probably the sputum positivity at the end of IP is more likely to be associated with poorly controlled DM status.

The effectiveness of DOTS therapy has been well established worldwide; however, a combination regimen is often a concern to evaluate the safety of a given drug. Pharmacokinetic interactions along with thorough knowledge of possible side effects will always enable a clinician to treat patients with anti-TB drugs more safely. The overall ADR in total patients was 70.9% including 92.0% and 69.9% in DM and non-DM patients, respectively. A few previous studies analyzed adverse events during anti-TB treatment in India, but the subjects were recruited from single location and sample sizes were small. Moreover, to date there are no published reports on incidence of adverse events during anti-TB treatment in DM and no-DM patients. The ADR incidences observed in this study are similar to previously published studies in Bangladesh, Nepal, and India [37–39]. Gholami et al. (2006) revealed 54.3% ADR incidences, associated with TB medications, in Iranian patients [40]. Presence of DM is significantly associated (OR: 3.578 95% CI: 1.114–11.494, *p* = 0.032) with anti-TB ADR, which may be attributed to the concomitant antidiabetic medications. However, we could not perform the subanalyses of DM pharmacotherapy in patients compared to no-DM patients. Lower plasma level of rifampicin has been reported in DM patients; however, the exact mechanism is yet to be elucidated [41, 42]. To our knowledge, no data exists concerning the incidence of ADRs in DOTS patients, particularly in those with DM. It would be mandatory to ascertain individuals having more risk of developing ADRs after initiating anti-TB treatment and they should be followed up by closer monitoring.

### 4.1. Strength and Limitations

These findings provided significant evidence and contributed to a better understanding and proper management in the course of TB among DM patients. Instead of medical records, we relied on laboratory tests to determine DM status. Diagnosis of DM was based on repeated glucose measurements rather than one-point estimation to avoid the misclassification of cases of DM due to a mixture of biological variation in blood glucose levels and measurement error. We referred newly diagnosed DM patients to the healthcare physician for DM management and also recorded the DM pharmacotherapy in this study. The side effects related to anti-TB drugs are inclusive of the entire treatment duration rather than those evolved in the initial phase of anti-TB therapy.

The results should be interpreted in the light of few limitations as the findings of this study are clearly not representative of all tuberculosis patients. We restricted our study to new and retreatment TB cases (category I and category II) only leaving other classes of TB patients. We did not include MDR TB patients; hence we lack the data on susceptibility to anti-TB drugs in TB patients complicated with DM. Further, the radiological interpretations were not obtained from most of the patients. Smear cultures were not collected as they are not performed routinely in TB clinics and the treatment outcome was mainly based on the sputum smear results. We could not assess the relationship between impaired glycaemia and diabetes and pulmonary TB. Information on severity of DM and their association with TB treatment outcome could not be evaluated in this study. This work was mainly confined in urban including slum area of south Delhi. Further prospective longitudinal interventional randomized studies covering larger sample including urban and rural populations of different subject group are necessarily recommended.

## 5. Conclusion

Active screening measures for DM are recommended in patients with TB which could improve the diagnosis and early management of DM complications. Treatment outcomes in patients with DM presence have been a subject of debate. Moreover, there is insufficient number of studies available in settings with high burdens of both diseases. There is further need of studying the effect of long-term evolution of DM control and associated complications on TB treatment outcome. Glycemic control should be strictly maintained, particularly, during the initial intensive phase for better outcome in patients with DM.

## Figures and Tables

**Table 1:** Definitions of TB treatment outcome.

Terms	Definitions
Cured	A PTB patient with bacteriologically confirmed TB at the beginning of treatment who was smear- or culture-negative in the last month of treatment and on at least one previous occasion

Treatment completed	A TB patient who has completed treatment without evidence of failure but with no record to show that sputum smear or culture results in the last month of treatment and on at least one previous occasion were negative, either because tests were not done or because results are unavailable

Treatment failed	A TB patient whose sputum smear or culture is positive at month 5 or later during treatment

Died	A TB patient who dies for any reason before starting or during the course of treatment

Lost to follow-up	A TB patient who did not start treatment or whose treatment was interrupted for 2 consecutive months or more

Not evaluated	A TB patient for whom no treatment outcome is assigned. This includes cases “transferred out” to another treatment unit as well as cases for which the treatment outcome is unknown to the reporting unit

Treatment success	The sum of *cured* and *treatment completed*

**Table 2:** Characteristics of enrolled patients at baseline.

Variables	TB with DM (*n* = 50)	TB without DM (*n* = 266)	*p* value
Male	27 (54.0)	148 (55.6)	0.831
Female	23 (46.0)	118 (44.4)	

Age (years) (mean ± SD)	44.04 ± 14.04	30.96 ± 12.89	<0.001^*∗∗∗*^

Family history of TB	05 (10.00)	38 (14.28)	0.417

Mean BMI (kg/m^2^) (mean ± SD)	19.81 ± 3.62	17.17 ± 3.26	0.307

*Marital status*			<0.001^*∗∗∗*^
Married	45 (90.0)	164 (61.6)	
Unmarried	02 (4.0)	99 (37.2)	
Widow/divorced/other	03 (6.0)	03 (1.12)	

*Religion*			0.595
Hindu	41 (82.0)	226 (84.9)	
Muslim	8 (16.0)	38 (14.3)	
Christian	1 (2.0)	2 (0.75)	

*Type of TB cases*			0.799
New	41 (82)	214 (80.4)	
Retreatment	09 (18)	52 (19.6)	

*Habitual risk *			
Alcohol	22 (44.0)	90 (33.8)	0.168
Smoking	15 (30.0)	56 (21.0)	0.164
Chewing	08 (16.0)	45 (16.9)	0.873

*Literacy level*			0.003^*∗∗*^
Illiterate	17 (34.0)	33 (12.4)	
Primary school	01 (2.0)	10 (3.8)	
Middle school	14 (28.0)	66 (24.8)	
High school	13 (26.0)	95 (35.7)	
Intermediate	04 (8.0)	44 (16.5)	
Graduate and professional degree	01 (2.0)	18 (6.8)	

*Sign and symptom*			
Cough	36 (72.0)	208 (78.2)	0.329
Loss of weight (more than 5 kg)	39 (78.0)	249 (93.6)	0.003^*∗∗*^
Anorexia	31 (62.0)	180 (67.7)	0.721
Evening rise in fever	28 (56.0)	165 (62.0)	0.405
Dyspnea	32 (64.0)	137 (51.5)	0.530
Chest pain	28 (56.0)	139 (52.2)	0.136
Hemoptysis	21 (42.0)	97 (36.5)	0.167

TB: tuberculosis; DM: diabetes mellitus; SD: standard deviation; data were analyzed using chi square test between TB with DM and TB without DM groups. *p* < 0.05 was considered as significant. ^*∗∗∗*^ *p* < 0.001; ^*∗∗*^ *p* < 0.01.

**Table 3:** Sputum conversion and treatment outcome of PTB patients with and without DM.

Variables	TB with DM (*n* = 50) *n* (%)	TB without DM (*n* = 266) *n* (%)	OR (95% CI)^$^	*p* value
PTB^#^	37 (74.0)	154 (57.9)	2.168 (1.102–4.265)	0.023^!^
EPTB^&^	13 (26.0)	112 (42.1)	1	

PTB patients (*n* = 36 in TB with DM group and *n* = 150 in TB without DM group)^*∗*^				
*Sputum status at 2 months*				
Sputum positive	10 (27.8)	37 (24.7)	1.222 (0.537–2.779)	0.633
Sputum conversion	26 (72.2)	113 (75.3)	1	
*Treatment outcomes*				0.428
Successful outcomes			1	
Cured	26 (72.2)	93 (62.0)		
Treatment completed	06 (16.7)	43 (28.7)		
Poor outcomes			1.176 (0.310–4.457)	
Default	02 (5.6)	02 (1.3)		
Failure	02 (5.6)	03 (2.0)		
Died	01 (2.8)	09 (6.0)		
Shifted to MDR	00	04 (2.7)		

^#^31 and 6 patients were sputum positive and sputum negative for AFB in TB with DM group. 109 and 45 patients were sputum positive and sputum negative for AFB in TB without DM group. ^*∗*^1 patient died before completion of IP in TB with DM group, 3 died, and 1 was shifted to MDR before completion of IP in TB without DM group.

^&^All patients with EPTB in both groups had successfully completed treatment.

^$^OR: odds ratio; calculated from binary logistic regression analysis; ^!^ *p* < 0.05 was considered as significant.

**Table 4:** Association of treatment outcomes and different variables in TB patients.

Variables	Poor outcome (*n* = 23)	Treatment success (*n* = 293)	*p* value
Sex			0.32
Male	15 (65.2)	160 (54.6)	
Female	08 (34.8)	133 (45.4)	

Category			<0.001^*∗∗∗*^
Category I	12 (52.2)	243 (82.9)	
Category II	11 (47.8)	50 (17.1)	

TB types			<0.001^*∗∗∗*^
PTB	23 (100)	168 (57.3)	
EPTB	00	125 (42.7)	

Family history of TB			0.94
Yes	03 (13.1)	40 (13.6)	
No	20 (86.9)	253 (86.3)	

Alcoholic history			0.08
Yes	12 (52.2)	100 (34.1)	
No	11 (47.8)	193 (65.9)	

Smoking history			0.04^*∗*^
Yes	09 (39.1)	62 (21.2)	
No	14 (60.8)	231 (78.8)	

Chewing history			0.51
Yes	05 (21.7)	48 (16.4)	
No	18 (78.2)	245 (83.6)	

ADR			0.74
Present	17 (73.9)	207 (70.6)	
Absent	06 (26.1)	86 (29.3)	

Cough			0.25
Present	20 (86.9)	224 (76.4)	
Absent	03 (13.1)	69 (23.5)	

Weight loss			0.02^*∗*^
Present	18 (78.3)	270 (92.1)	
Absent	05 (21.7)	23 (7.8)	

Anorexia			0.09
Present	19 (82.6)	192 (65.5)	
Absent	04 (17.4)	101 (34.5)	

Fever			0.008^*∗∗*^
Present	20 (86.9)	173 (59.1)	
Absent	03 (13.0)	120 (40.9)	

Dyspnea			0.01^*∗*^
Present	18 (78.3)	151 (51.5)	
Absent	05 (21.7)	142 (48.4)	

Chest pain			0.04^*∗*^
Present	17 (73.9)	150 (51.2)	
Absent	06 (26.1)	143 (48.8)	

Hemoptysis			0.13
Present	12 (52.2)	106 (36.2)	
Absent	11 (47.8)	187 (63.8)	

DM			0.42
Present	05 (21.7)	45 (15.4)	
Absent	18 (78.3)	248 (84.6)	

Poor outcome: default, death, failure, and regimen changed; treatment success: cured and treatment completed; PTB: pulmonary tuberculosis; EPTB: extrapulmonary tuberculosis; DM: diabetes mellitus; ADR: adverse drug reaction; data was analyzed using chi square test. *p* ≤ 0.05 was considered as significant. ^*∗∗∗*^ *p* < 0.001, ^*∗∗*^ *p* < 0.01, and ^*∗*^ *p* < 0.05.

**Table 5:** Association of clinical manifestations with sputum positivity and treatment outcomes among TB patients by multivariate analyses.

Variables	Sputum positive > 60 days OR (95% CI)	Poor outcome OR (95% CI)
DM	0.633 (0.206–1.949)	0.714 (0.155–3.279)
Men	1.284 (0.327–4.430)	0.312 (0.055–1.762)
Age	0.992 (0.957–1.029)	0.960 (0.904–1.020)
Category	1.685 (0.647–4.391)	0.838 (0.243–2.888)
BMI	0.914 (0.794–1.052)	1.185 (0.970–1.447)
TB history	1.797 (0.491–6.582)	2.591 (0.260–25.821)^*∗*^
Alcohol intake	0.880 (0.280–2.764)	0.674 (0.177–2.558)
Smoking	1.811 (0.673–4.877)	0.752 (0.236–2.390)
Chewing	0.689 (0.252–1.883)	0.778 (0.241–2.543)
ADR incidence	1.797 (0.491–6.582)^*∗*^	0.642 (0.187–2.207)
Weight gain	0.914 (0.141–5.915)	0.708 (0.059–8.521)
Anorexia	1.165 (0.060–22.585)	0.558 (0.048–3.124)
Fever	2.176 (0.321–14.734)^*∗*^	0.814 (0.093–2.155)
Dyspnea	0.138 (0.010–1.902)	1.973 (0.337–5.871)^*∗*^
Chest pain	0.090 (0.010–0.847)	1.370 (0.485–6.143)
Hemoptysis	1.582 (0.062–0.551)	0.813 (0.291–2.275)

OR: odds ratio; CI: confidence interval; BMI: body mass index; ADR: adverse drug reaction; DM: diabetes mellitus. All independent variables were analyzed using multiple logistic regression analysis to calculate the odds ratio. The OR presented is adjusted for age, gender, and BMI in logistic regression analysis. ^*∗*^ *p* ≤ 0.05 was considered as significant.

**Table 6:** Total ADRs collected from all patients.

ADR	TB with DM (*n* = 50)		TB without DM (*n* = 266)		*p* value
	ADR incidence *n* (%)	Onset time, days (median, IQR)	ADR incidence *n* (%)	Onset time, days (median, IQR)	
Nausea and vomiting	13 (26.0)	12 (7–26)	50 (18.80)	14 (5–32)	0.242
Rashes	12 (24.0)	16 (10–29)	62 (23.31)	13 (5–45)	0.916
Peripheral neuropathy	18 (36.0)	17 (7–50)	62 (23.31)	15 (10–39)	0.058
Liver injury	11 (22.0)	25 (17–54)	48 (18.04)	32 (25–65)	0.510
Restlessness	21 (42.0)	10 (5–25)	48 (18.04)	08 (5–21)	<0.001^*∗∗∗*^
GI problem^%^	07 (14.0)	12 (5–40)	23 (8.65)	16 (11–32)	0.236
Hypoglycemia	02 (4.0)	06 (3–10)	00	00	0.001^*∗∗∗*^
Joint pain^#^	07 (14.0)	20 (11–45)	32 (12.03)	14 (10–55)	0.698
Drowsiness	05 (10.0)	11 (5–27)	41 (15.41)	08 (3–21)	0.319
Back pain	07 (14.0)	37 (15–55)	09 (3.38)	22 (12–51)	0.002^*∗∗*^
Feet pain	05 (10.0)	30 (25–39)	06 (2.25)	21 (18–45)	0.006^*∗*^
Body ache	05 (10.0)	10 (7–18)	11 (3.48)	18 (10–24)	0.083
Blurring vision	01 (2.0)	60	03 (1.12)	55 (42–64)	0.613
Other nervous system disorders^$^	20 (40.0)	08 (03–18)	74 (23.4)	06 (15–50)	0.084

^%^Loss of appetite and diarrhea; ^$^sleep disorders, headache, irritability, and vertigo; ^#^arthralgia and Achilles pain; ADR: adverse drug reaction; IQR: interquartile range; data were analyzed using chi square test. ^*∗*^ *p* ≤ 0.05 was considered as significant. ADR reported by TB with DM patients = 46; ADR reported by TB without DM patients = 178. ^*∗∗∗*^ *p* < 0.001, ^*∗∗*^ *p* < 0.01, and ^*∗*^ *p* < 0.05.

**Table 7:** ADR incidence in different categories of TB patients with and without DM.

ADRs	Category I (*n* = 255)			Category II (*n* = 61)			
	TB with DM (*n* = 41)	TB without DM (*n* = 214)	*p* value^1^	TB with DM (*n* = 09)	TB without DM (*n* = 52)	*p* value^2^	*p* value^3^
	ADR incidence, *n* (%)	ADR incidence, *n* (%)		ADR incidence, *n* (%)	ADR incidence, *n* (%)		
Nausea and vomiting	11 (26.8)	38 (17.7)	0.177	02 (22.2)	12 (23.07)	0.955	0.775
Rashes	10 (24.4)	43 (20.1)	0.534	02 (22.2)	19 (36.5)	0.404	0.890
Peripheral neuropathy	14 (34.1)	45 (21.0)	0.068	04 (44.4)	17 (32.7)	0.493	0.560
Liver injury	07 (17.1)	32 (15.0)	0.730	04 (44.4)	16 (30.8)	0.420	0.073
Restlessness	15 (36.6)	36 (16.8)	0.004^*∗*^	06 (66.6)	12 (23.1)	0.008^*∗*^	0.098
GI problem	05 (12.2)	18 (8.4)	0.438	02 (22.2)	05 (9.6)	0.273	0.432
Hypoglycemia	01 (2.4)	00	0.022^*∗*^	01 (11.1)	00	0.015^*∗*^	0.229
Joint pain	05 (12.2)	19 (8.9)	0.505	02 (22.2)	13 (25.0)	0.858	0.432
Drowsiness	05 (12.2)	31 (14.5)	0.700	00	10 (19.2)	0.150	0.269
Back pain	05 (12.2)	07 (3.3)	0.013^*∗*^	02 (22.2)	02 (3.9)	0.040^*∗*^	0.432
Feet pain	04 (9.7)	06 (2.8)	0.036^*∗*^	01 (11.1)	00	0.015^*∗*^	0.902
Body ache	04 (9.7)	07 (3.3)	0.061	01 (11.1)	04 (7.7)	0.730	0.902
Blurring vision	01 (2.4)	03 (1.4)	0.624	00	00	00	0.636
Other nervous system disorders	16 (39.0)	52 (24.3)	0.051	04 (44.4)	22 (42.3)	0.905	0.764

^1^Data compared between TB with DM and TB without DM group in category I patients.

^2^Data compared between TB with DM and TB without DM group in category II patients.

^3^Data compared between TB with DM patients between category I and category II patients.

ADR: adverse drug reaction; TB: tuberculosis; DM: diabetes mellitus; all data has been analyzed by chi square test; ^*∗*^ *p* ≤ 0.05 was considered as significant.

**Table 8:** Number of adverse reactions recorded in DM and non-DM TB patients.

Number of ADRs reported	TB with DM *n* = 50	TB without DM *n* = 266	*p* value
No ADRs	04 (8.0)	89 (33.46)	<0.001^*∗∗∗*^
One	06 (12.0)	47 (17.67)	0.325
Two	08 (16.0)	55 (20.67)	0.448
Three	13 (26.0)	40 (15.04)	0.057
Four	05 (10.0)	28 (10.53)	0.911
Five	07 (14.0)	04 (1.50)	<0.001^*∗∗∗*^
More than five	07 (14.0)	03 (1.13)	<0.001^*∗∗∗*^

ADR: adverse drug reaction; TB: tuberculosis; DM: diabetes mellitus; values in parenthesis are expressed in percentage; values were compared by using chi square test. *p* ≤ 0.05 was considered as significant. Statistical calculation was performed by using chi square test between the two groups. ^*∗∗∗*^ *p* < 0.001.

**Table 9:** Multivariate analysis showing factors associated with TB treatment adverse effect.

Variables	OR (95% CI)	*p* value
Age	0.970 (0.940–1.001)	0.062
Male	2.013 (0.906–4.473)	0.086
Category I cases	2.165 (1.004–4.670)	0.049^*∗*^
PTB	1.071 (0.555–2.065)	0.838
Married	1.618 (0.804–3.258)	0.178
Hindu	1.131 (0.498–2.567)	0.769
BMI	0.965 (0.874–1.065)	0.478
Joint family	0.725 (0.359–1.462)	0.369
Alcoholic history	0.942 (0.427–2.078)	0.883
Smoking history	0.853 (0.411–1.768)	0.669
Chewing history	0.617 (0.275–1.385)	0.242
Family TB history	0.451 (0.184–1.105)	0.081
Presence of DM	3.578 (1.114–11.494)	0.032^*∗*^
Cough	1.104 (0.426–2.864)	0.839
Weight loss	1.326 (0.411–4.276)	0.637
Anorexia	0.309 (0.059–1.607)	0.163
Fever	1.276 (0.383–4.245)	0.691
Dyspnea	0.733 (0.278–1.934)	0.531
Chest pain	0.857 (0.279–2.633)	0.788
Hemoptysis	1.069 (0.564–2.026)	0.839

OR: odds ratio; TB: tuberculosis; DM: diabetes mellitus.

All independent variables were analyzed using multiple logistic regression analysis. ^*∗*^ *p* < 0.05 was considered as significant. The OR presented is adjusted for age, gender, and BMI in logistic regression analysis.
